# Comparison of Prior Setting Methods for Multilevel Model Effect Estimation Based on Small Sample Imbalanced Nested Data in Bayesian Framework

**DOI:** 10.1155/2022/2726602

**Published:** 2022-11-14

**Authors:** Guangming Li, Like An

**Affiliations:** ^1^Key Laboratory of Brain, Cognition and Education Sciences, South China Normal University, Ministry of Education, Guangzhou, China; ^2^School of Psychology, Center for Studies of Psychological Application, and Guangdong Key Laboratory of Mental Health and Cognitive Science, South China Normal University, Guangzhou, China

## Abstract

In the fields of education and psychology, nested data with small samples and imbalances are very common. Bauer et al. (2008) first proposed adjusting the traditional multilevel model to analyze the small sample imbalanced nested data (SSIND). In terms of parameter estimation, the Bayesian method shows the possibility of providing unbiased estimation when the sample size is small. This study proposes that the Bayesian method should be used to analyze the SSIND. This study explores the performance of different treatment effects and nesting effects estimation methods in the multilevel model based on the Bayesian method that performs well in the case of small samples, to provide an appropriate and scientific method reference for the subsequent analysis of the model. Two prior setting methods are compared for multilevel model effect estimation based on a small sample of imbalanced nested data in the Bayesian framework. Two prior setting methods are gamma prior setting method and uniform prior setting method. The research results show that when the treatment condition ICC is small (0.05), the bias and RMSE values of the parameter estimation by the gamma prior setting method are larger and the performance is unstable, while the bias and RMSE values of the parameter estimation by the uniform prior setting method are smaller and the performance is relatively stable, so the uniform prior setting method is recommended; when the treatment condition ICC is large (0.15), the bias and RMSE values of the parameter estimation by the uniform prior setting method are larger and the performance is unstable, while the bias and RMSE values of the parameter estimation by the gamma prior setting method are smaller and the performance is relatively stable, so the gamma prior setting method is recommended; when the treatment condition ICC is between 0.05 and 0.15, both prior setting methods have similar effects. Furthermore, when the number of treatment groups is small (8), the gamma prior setting method is recommended; when the number of treatment groups is large (16), the uniform prior setting method is recommended; when the number of treatment groups is between 8 and 16, both prior setting methods have similar effects. Summarily, when we choose which prior setting method to use for the SSIND, we must consider the interaction between the ICC and the number of treatment groups.

## 1. Introduction

In social science, many research questions are embodied in multilevel or multilayered data structures. For example, students in these classes evaluate their teachers. Different teachers teach different classes of students, and the students are nested in the classes. The data structure of this design is multilevel or multilayer. However, in some cases, the population that researchers are interested in may be small, and it will be difficult to collect many samples. Therefore, researchers must collect a small sample of data with different subjects nested in different imbalanced groups, which is called “Small Sample Imbalanced Nested Data” (SSIND) and is very common in the fields of education and psychology. Bauer et al. [1] found that although certain studies on multilevel models and nested data have been conducted, the methodological research on nested data with imbalances in small samples has only begun recently. For this type of data, the traditional analysis method is to ignore the imbalanced structure of the data and use a linear regression model to analyze it or to treat it as balanced nested data and use a traditional multilevel model to analyze it. These two methods cannot correctly explain the similarity among individuals in each treatment group, nor can they correctly estimate the treatment effects that researchers are concerned about. Therefore, using a multilevel model that conforms to the data for analyzing is crucial to the accuracy of the parameter estimation results [2–6].

For the nested data in common educational research, traditional regression analysis has two treatment methods.

Firstly, all higher level variables are treated as first level variables and the data are analyzed directly at the individual student level. The problem with this method is that the class variables have the same effect on students in the same class without distinguishing the effects of the class on the students. It is unreasonable to assume that students in the same class are independent of each other. It is also unreasonable for students in the same class to make the same assumption.

Secondly, the observations of the first level are merged directly into the observations of the second level and then directly the class is analyzed. The main problem with this is that information about the differences among individual students in the class is lost. In practice, this part of the variation may account for a large part of the total variation.

Based on the above discussion, these two methods have one thing in common: they do not consider the characteristics of data stratification. The first method will infer the analysis results of microunit data (student level) to the macro-unit (class level). It is guilty of the “ecological fallacy”; the second method will commit the “reductionism fallacy” (reductionism fallacy) when inferring the analysis results of the macro-unit (class level) to the micro-unit (student level). This is a limitation of the traditional regression analysis methods when analyzing data with a nested structure.

The traditional linear regression model assumes that there is a straight line relationship among variables; the variables as a whole obey a normal distribution; the variance is homogeneous, and the random errors among individuals are independent of each other. The first two assumptions are easy to guarantee, but the homogeneity of variances, especially the assumption that the random errors among individuals are independent of each other, is difficult to meet. It is that students in different classes can be assumed to be independent of each other, but students in the same class are affected by the same class variable; thus, it is difficult to guarantee mutual independence. Therefore, in the analysis, the error in the traditional regression analysis should be decomposed into two parts: one is the error caused by the difference among the individuals at the first level and the other is the error caused by the difference at the second level. It can be assumed that the measurement errors among individuals at the first level are independent of each other, and the errors caused by classes at the second level are independent of each other among different classes. This is the core of the multilevel analysis.

Due to the advantages of decomposition errors, the development of multilevel linear models began to mature in the mid to late 1980s. This model has different titles in different disciplines. In educational research, it is named the hierarchical linear model (HLM); in biostatistics, it is called the mixed-effects model and random effects model; in econometrics, it is called the random coefficient regression model; it is called a covariance component model in the statistical literature.

In addition, in terms of parameter estimation, the classic parameter estimation method mainly uses the maximum likelihood estimation method. However, when the number of high-level units is small or the data structure is imbalanced, the maximum likelihood estimation has certain shortcomings in the estimation accuracy [7]. Although some studies have improved the parameter estimation methods for nested data under the framework of multilevel models, such as various small sample correction methods, these estimation methods can modify the *p* value of fixed effects and confidence interval estimation after adjusting the standard errors and degrees of freedom [8, 9]. However, when estimating the variance of the second-level nesting effect in the model, these methods do not solve the uncertainty problem in the estimation, and the nesting variance cannot be estimated correctly. Recently, the Bayesian method has shown the possibility of providing an unbiased estimate when the sample size is small, but related research is still very scarce, particularly in the selection of prior distributions, and there are differences among researchers [10, 11]. There are several major papers in the general Bayesian multilevel literature, such as Depaoli and Clifton [12]; Fang et al. [13]; Helm [14]; McNeish [15]; van Erp et al. [16]; and Zitzmann et al. [17]. While these papers may focus on general multilevel model specifications different from the one used in the current paper, they offer valuable insight into the impact of prior specifications and Bayesian estimation but they do not focus on the SSIND.

Therefore, this article uses simulation research based on the research of common multilevel models and uses the Bayesian method that performs well in the case of small samples and explores the different treatment effects and nesting effect estimation methods in multilevel models. Furthermore, in terms of the prior setting of parameters, it provides some suggestions for empirical researchers to analyze the imbalanced nested data of small samples.

Since Bauer et al. [1] proposed a new method for analyzing imbalanced nested data, some studies have been conducted to explore whether the model has advantages over other models, but most of the research results show that the model performs better [2–6]. In addition, the population that the researchers are interested in may be small, so collecting large numbers of samples may be challenging. This research may also be exploratory. The researchers do not intend to collect a large sample size due to practical problems such as financial constraints or difficulty in recruiting many participants. Nested data with a small number of treatment groups are very common. For example, recruiting many higher level units (such as schools or hospitals) to participate in research requires a lot of costs. Moreover, some populations may only be sparsely distributed and have a small number of people, so it is difficult to collect a large number of samples (for example, the number of schools set up for deaf students in the United States is small). Therefore, nested data with small samples and imbalances are very common.

In the past ten years, some simulation studies have used various methods to treat the small sample characteristics of imbalanced nested data, such as the model proposed by Bauer et al. [1] (MLMs; e. g., [2, 4, 6, 18]). These studies usually show that models with about 20 to 40 treatment groups can exhibit ideal properties (for example, consistency), and if the Kenward-Roger method, which corrected the restrictive maximum likelihood method (REML), is used for parameter estimation, only 10 to 20 treatment groups can maintain the ideal statistical attributes. But, other studies advocate the use of Bayesian methods when the number of treatment groups is small [10, 18–21], especially in estimating the variance component (when the number of treatment groups is small, the likelihood method can be difficult to estimate the variance component) [22]. Gelman [23] obtained an unbiased estimate of the intercept variance in the case of only three treatment groups through an application example, in the case of carefully choosing the prior distribution. The following literature presents related research on the analysis of SSIND.

Baldwin et al. [2] used simulation studies to compare five models for analyzing small sample imbalanced nested data, namely, linear regression models, models that treat each group of treatment conditions as fixed effects, and traditional multilevel models, setting the residuals of the first level under the treatment conditions and the control conditions in the model proposed by Bauer et al. [1] as equal and unequal models. The study also compared the performance of three methods for calculating degrees of freedom: the between-within method, the Satterthwaite approximation method, and the Kenward-Roger method. The results found that the model proposed by Bauer et al. [1] had a better Type I error rate when estimating fixed effects than the other three models. Whether the model is homogeneous or not does not affect the Type I error rate. The study also found that at least 8 treatment groups are required. Also, it is best to have 16 or more to maintain the nominal Type I error rate. In addition, the Satterthwaite method for calculating degrees of freedom is superior to the between-within method, and the effect is similar to the Kenward-Roger method. Although the estimation of the treatment effects is unbiased when the model is homogeneous or heterogeneous, when the value is large and the number of treatment groups and the number of subjects in each group are small, the estimate of the second-level variance component is biased.

Korendijk et al. [4] evaluated three models for analyzing the SSIND, including the model proposed by Bauer et al. [1]; a multilevel model that treats each subject in the control group as a treatment group, and a multilevel model that treats the control group as a large treatment group. The results show that when the number of treatment groups and the intragroup correlation coefficient under treatment conditions are very small (*c* = 10, *ρ* = 0.05), the variance component will have a negative estimate. The multilevel models that treat the control group as a large-treatment group are more likely to produce unobserved solutions. In addition, the model proposed by Bauer et al. [1] and the multilevel model that treats each subject in the control group as a treatment group have roughly the same deviations in the estimated parameters, although the structure of the variance component specified by the latter is wrong.

Sanders [6] uses four methods to analyze small sample imbalanced nested data, including a multilevel model that treats the control group as a large-treatment group, and divides the control group into several groups. A multilevel model has the same number of each group as the treatment group. The other two models treat the subjects in the control group as a treatment group: one model is a random intercept with a fixed slope, and the other model is a fixed intercept with a random slope. The study also compared two methods for calculating degrees of freedom, namely, the between-within method and the Kenward-Roger method. The results of the study are similar to those of Baldwin et al. [2]; the Kenward-Roger method is better than the between-within method; furthermore, increasing the number of treatment groups can improve the statistical test power compared to increasing the number of subjects in each group. In addition, models with fixed intercepts and random slopes perform better than the other models in most cases.

Candlish et al. [3] used a simulation study to compare the performance of six models when analyzing the SSIND. The results showed that when the number of treatment groups (3–6) and the number of subjects in each group (5–10) and the ICC (Intra-class Correlation Coefficient) (≤0.05) are small, there is no optimal model.

The above research shows that under most conditions, the model proposed by Bauer et al. [1] performs best when analyzing the SSIND. However, when the sample size is small or the intragroup correlation coefficient is small under the treatment conditions, the performance of the model is not sufficiently good [3]. McNeish [24] pointed out that about 33% of the growth model, 20% of the multilevel model, 40% of the meta-analysis, and 30% of the random control experiment data have small sample problems. In addition, when the sample size is relatively small and the variance structure of the data is complex, the inference of the variance component and fixed effects is very complicated due to the uncertainty of the true value of the variance component. Even if researchers use the model proposed by Bauer et al. [1] to analyze such data, the estimation methods used are often common maximum likelihood estimation or restrictive maximum likelihood estimation methods. After adjusting the standard errors and degrees of freedom, these estimation methods can revise the *p* value and confidence interval estimates of the fixed effects [8, 9]. However, these adjustments did not directly solve the uncertainty of estimating the variance component, and it is impossible to estimate the variance component correctly. The following literature presents some research studies on the comparison of parameter estimation methods under the framework of multilevel models.

Baldwin and Fellingham [10] compared the performance of the likelihood method and the Bayesian method in the case of a small sample based on the model proposed by Bauer et al. [1]. The results show that for fixed effects, the two estimation methods perform well in terms of *bias*, efficiency, and confidence interval coverage; for variance components, the carefully selected gamma prior Bayesian method has more deviation but higher estimation efficiency compared to the restrictive maximum likelihood method. In addition, in the case of a small sample, the inference of the variance component is very sensitive to the choice of the prior distribution.

Based on a multilevel model, McNeish and Stapleton [5] compared the performance of the full maximum likelihood method and the restrictive maximum likelihood method in the case of a small sample. The results show that for continuous outcome variables, the restrictive maximum likelihood estimation method is better than the maximum likelihood method in estimating the variance components, and the Kenward-Roger method, which adjusted the REML, can improve the problem of underestimated fixed effect standard errors.

van de Schoot et al. [11] systematically reviewed the application of the Bayesian method in the field of psychology in the past 25 years and found that this method only accounts for 3% of the simulation research of multilevel models. The simulation research of the multilevel model may exist in other fields [18], but this also shows that the relevant research using the Bayesian method to estimate that the multilevel model is indeed lacking. In a small number of existing studies, the Bayesian method has shown the possibility of providing unbiased estimates when the sample size is small [7, 10, 25–27].

In summary, in terms of parameter estimation, the Bayesian method shows the possibility of providing unbiased estimation when the sample size is small. This study proposes that the Bayesian method should be used to analyze the SSIND. However, few scholars discuss the prior setting of Bayesian methods based on SSIND, which is insufficient. In fact, the inference of variance components is very sensitive to the selection of prior distribution in small samples. According to Baldwin and Fellingham [10], gamma prior setting and uniform prior setting are the two most common prior settings. But, Baldwin and Fellingham [10] did not compare the performance of the two prior setting methods. This study will compare the performance of two prior setting methods (i.e., gamma prior setting method and the uniform prior setting method) for multilevel model effect estimation based on a small sample of imbalanced nested data in a Bayesian framework. Meanwhile, this study will also explore the performance of different treatment effects and nesting effects estimation methods in the multilevel model based on the Bayesian method that performs well in the case of small samples, to provide an appropriate and scientific method reference for the subsequent analysis of the model.

## 2. Methods

### 2.1. Truth Model

Using a self-made R language program, refer to the multilevel model proposed by Bauer et al. [1] that is consistent with the data structure of small sample imbalanced nested data and generate the following truth model:

(1) $$\begin{array}{c} Y_{ij}=b_{0}+b_{1}X_{ij}+u_{j}Z_{j}+e_{ij}\text{.} \end{array}$$

There are 3 × 4 × 3 × 2 = 72 conditions. Refer to Tessler [28] to set the mean value of observations under the control condition to 2 and the treatment effect under the treatment condition to 0.5. Refer to Baldwin and Fellingham [10] to set the error under the control condition to obey a normal distribution with a mean value of 0 and a variance of 0.27, and set the total error under the treatment conditions to obey a normal distribution with a mean value of 0 and a variance of 0.46. The nesting effect under the treatment conditions is set as $\sqrt{\rho }z_{ij}$, and the error of the individual level under the treatment condition is set as $\sqrt{1-\rho }z_{ij}$, and *ρ* = the variance of the second level/(the variance of the second level + the variance of the first level).

### 2.2. Independent Variables

Since the common imbalanced nested data are mostly small samples, the manipulation conditions of this study are also set by referring to the situation surrounding small samples in previous studies.

The number of treatment groups was 8, 12, and 16. Baldwin et al. [2] set the number of treatment groups to four levels of 2, 4, 8, and 16, and Candlish et al. [3] set it to four levels of 3, 6, 12, and 24. In this study, the setting of the number of treatment groups on the basis of the predecessors is all about the small sample situation.

The number of participants in each group was 5, 10, 15, and 20. Baldwin et al. [2] set the number of participants in each group to three levels of 5, 15, and 30, and Candlish et al. [3] set it to four levels of 5, 10, 20, and 30. Based on the predecessors, the number of participants in each group is set around a small sample situation in this study.

The ICC under the treatment conditions is 0.05, 0.1, and 0.15. Baldwin et al. [2] set the ICC under treatment conditions to five levels of 0, 0.05, 0.1, 0.15, and 0.3. In addition, in a few cases, the intragroup correlation coefficient under treatment conditions is greater than 0.3 [29–32]. Therefore, in this study, ICC was set to three levels of 0.05, 0.1, and 0.15 under the treatment conditions.

The estimation method: this study adopts the Bayesian method and sets uniform prior and gamma prior for *σ*_*U*_^2^ under its framework. Among them, referring to Baldwin and Fellingham [10]; the uniform prior is set as the following: *σ*_*U*_^2^ ~ *U*(0,0.23),*σ*_*e*_*C*__^2^ ~ *U*(0,0.69), an d *σ*_*e*_*U*__^2^ ~ *U*(0,0.69); the gamma prior is set as the following: *σ*_*U*_^2^ ~ *G*(13,0.03),*σ*_*e*_*C*__^2^ ~ *G*(13,0.03), an d,*σ*_*e*_*U*__^2^ ~ *G*(9,0.03).

### 2.3. Fixed Variables

Refer to Baldwin and Fellingham [10] to set the prior mean of the observations under the control conditions to a normal distribution with a mean of 3 and a variance of 2.25. Set the prior of the treatment effect under the treatment conditions to a normal distribution with a mean value of 0 and a variance of 1. Set the prior of nesting effects to a normal distribution with a mean of 0 and a variance of *σ*_U_^2^. With reference to Stice et al. [33], the prior of the total error under the treatment conditions is set to a normal distribution with a mean of 2.93 and a variance of 0.46.

### 2.4. Evaluation Index

Convergence rate is as follows:

(2) $$\begin{array}{c} rate=\frac{r}{R}\text{,} \end{array}$$

where *r* represents the number of convergences of the model and *R* represents the number of repetitions.

95% confidence interval coverage of true value is as follows:

(3) $$\begin{array}{c} cover=\frac{\left\{{\overset{\wedge }{X}}_{r}^{low}<X_{r}<{\overset{\wedge }{X}}_{r}^{up}\right\}}{R}\text{.} \end{array}$$

*R* represents the number of repetitions, ${\overset{ˇ}{X}}_{r}$ represents the parameter to be estimated, and *X*_*r*_ represents the true value of the parameter to be estimated.

(4) $$\begin{array}{c} Bias=\frac{\sum_{r=1}^{R}\left({\overset{\wedge }{X}}_{r}-X_{r}\right)}{R}\text{,}\\ RMSE=\sqrt{\frac{\sum_{r=1}^{R}{\left({\overset{\wedge }{X}}_{r}-X_{r}\right)}^{2}}{R}}\text{.} \end{array}$$

### 2.5. Research Process and Tools

The analysis tool adopts *R* software and *JAGS* software. First, use R software to generate 1 batch of simulated data under each combination of “number of treatment groups × number of subjects in each group × ICC under treatment condition” and then use two types of Bayesian methods. The prior setting method estimates the treatment effect and the variance of the nesting effect of each batch of data, and the final loop estimates 1000 times. The M-H sampling algorithm is used when fitting a multilevel Bayesian model to avoid slow mixing when estimating the variance, especially when the variance is small. Therefore, to obtain a more accurate estimation result, a longer Markov chain is required. Furthermore, this research refers to Baldwin and Fellingham [10] to choose 50,000 iterations and burn in 10,000 times. There were three Markov chains. After the Markov chain reaches a stable distribution, one sample is taken every 10th, and finally 4000 valid samples are obtained. The posterior distribution is composed, and the posterior estimation of the parameters is carried out accordingly. The analysis results were carried out in Excel 2010. Interested readers can request the code from the corresponding author.

## 3. Results

### 3.1. Estimated Accuracy Results of Treatment Effects and Nesting Effects for the SSIND under All Operating Conditions

The estimation accuracy results of treatment effects and nesting effects are presented under all operating conditions. The *bias* and root-mean-square error (RMSE) of the model convergence rate, confidence interval coverage, the treatment effect, and nesting effect estimation under 72 operating conditions are shown in Tables 1–3.

According to Tables 1–3, summarize and present the estimation accuracy results of treatment effects and nesting effects under all operating conditions (see Table 4).

According to Table 4, on the whole, in terms of convergence rate, the average value of the treatment effect under the gamma prior condition is 97.4%, the median value is 97.5%, the minimum value is 96.3%, and the maximum value is 98.2%. The average value of the treatment effect under the uniform is 97.0%, the median is 96.9%, the minimum is 96.0%, and the maximum is 98.2%. The mean value of the nesting effect under the gamma prior condition is 97.8%, the median value is 97.8%, the minimum value is 96.7%, the maximum value is 99.1%, the mean value of the nesting effect under the uniform prior condition is 99.0%, the median value is 99.0%, the minimum value is 98.1%, and the maximum value is 100%. Under the two prior conditions, the convergence rate is more than 96% and relatively concentrated, and the performance is very good, but the convergence rate of the gamma prior treatment effect is generally more stable than the uniform prior, and the convergence rate of the nesting effect is generally not as stable as the uniform prior.

In terms of confidence interval coverage, the mean value of the treatment effect under the gamma prior condition is 93.04%, the median value is 92.9%, the minimum value is 91.27%, and the maximum value is 95.17%. The mean value of the treatment effect under the uniform prior condition is 96.81%, the median is 96.51%, the minimum is 94.45%, and the maximum is 99.17%. The mean value of the nesting effect under the gamma prior condition is 89.49%, the median value is 93.14%, the minimum value is 57.58%, and the maximum value is 97.96%. The mean value of nesting effects under uniform prior conditions is 93.22%, the median value is 94.34%, the minimum value is 80.16%, and the maximum value is 97.46%. The coverage of the treatment effect under the two prior conditions is above 91%, which is very good, but the coverage of the gamma prior treatment effect is generally not as stable as the uniform prior. In addition, the coverage of the nesting effect under the two prior conditions is above 89%, which is excellent. However, the coverage of the two prior nesting effects is very low. Among them, the minimum nesting effect coverage rate under the gamma prior condition is 57.58%, and the minimum nesting effect coverage rate under the uniform prior condition is 80.16%. This is when the number of treatment groups is 16, the trial number of each group is 20, and the ICC is 0.05 under the treatment conditions. Combining the coverage of the two prior nesting effects under other conditions, it can be seen that ICC under the treatment conditions can affect the confidence interval coverage of the nesting effect more than the number of treatment groups and the number of subjects in each group. Moreover, with the increase of ICC under the treatment conditions, the coverage rate of the gamma prior nesting effect is higher than that of the uniform prior.

In terms of *bias*, the estimation accuracy of treatment effects under the condition of the gamma prior is higher than that of the uniform prior. Among them, the mean value of the treatment effect under the gamma prior condition is −69, the median value is −56, the minimum value is −171, and the maximum value is −6. The mean value of the treatment effect under the uniform prior condition is −101, the median value is −76, the minimum value is −301, and the maximum value is −23. The mean value of the nesting effect under the gamma prior condition is −143, the median value is −147, the minimum value is −398, the maximum value is 59, the mean value of the nesting effect under the uniform prior condition is 485, and the median value is 357. The minimum value is −291, and the maximum value is 1979. For the treatment effect, both prior conditions are underestimated; for the nesting effect, the uniform prior condition is mostly overestimated, and the underestimation occurred when the ICC is 0.05 under the treatment condition, and both underestimation and overestimation occur under the gamma prior condition when the ICC is 0.15 under the treatment conditions. In addition, as the number of treatment groups or the number of subjects in each group and ICC increases, compared with the uniform prior, the *bias* value of the treatment effect and nesting effect estimated by the gamma prior is smaller, and the change is also smaller. It is estimated that the result is more stable, indicating that the gamma prior estimation result is better than the uniform prior estimation result.

In terms of the RMSE, the estimation accuracy of the nesting effect under the gamma prior condition is lower than that of the uniform prior, and the estimation accuracy of the treatment effect is similar to that of the uniform prior. Among them, the average value of the treatment effect under the gamma prior condition is 79, the median value is 78, the minimum value is 46, and the maximum value is 125. The average value of the treatment effect under the uniform prior condition is 79, the median value is 77, and the minimum value is 45, and the maximum value is 123. The mean value of the nesting effect under the gamma prior condition is 30, the median value is 29, the minimum value is 25, and the maximum value is 40. The mean value of the nesting effect under the uniform prior condition is 65, the median value is 47, the minimum value is 26, and the maximum value is 198. In addition, as the number of treatment groups or the number of subjects per group and ICC increases, compared with the uniform prior, the estimated RMSE value of the treatment effect and the nesting effect of the gamma prior is smaller, and the change is also smaller. It is estimated that the result is more stable, indicating that the gamma prior estimation result is better than the uniform prior estimation result.

### 3.2. The Estimation Accuracy Results of Treatment Effects and Nesting Effects for the SSIND under Different Treatment Groups

**Figure 1:**
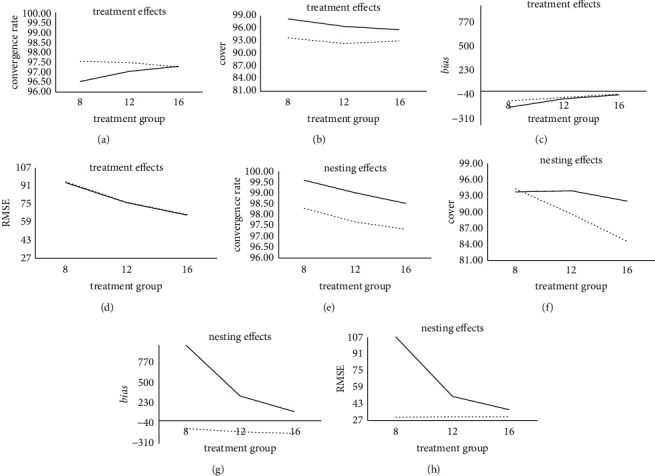
Comparison of estimation results of treatment and nesting effects for the SSIND under different treatment groups. Note: the dotted line represents the estimation result of the gamma prior, and the solid line represents the estimation result of the uniform prior.

According to Tables 1–3, under the condition of a different number of treatment groups, the results of treatment effect and nesting effect estimation are different. The result is shown in Figure 1.

As the number of treatment groups decreases, in terms of convergence rate, the convergence rate of the uniform prior treatment effects decreases slightly, but the convergence rate of the gamma prior treatment effects has been stable at a high level. In addition, the convergence rate of the two prior nesting effects has been improved, but the convergence rate of the uniform prior nesting effect is generally higher than that of the gamma prior. In terms of coverage, the coverage of treatment effects and nesting effects under gamma prior conditions has increased, but the coverage of uniform prior treatment effects and nesting effects has been stable at a higher level and more stable.

In terms of *bias*, the estimation accuracy of the treatment effect of the two prior setting methods is stable at a relatively high level. However, the *bias* value when the treatment effect is estimated by the gamma prior is generally higher than the *bias* value under the uniform prior condition, and the estimation result is more stable. In addition, the uniform prior has a significant reduction in the estimation accuracy of the nesting effect, and the gamma prior has a slight increase in the estimation accuracy of the nesting effect. However, the *bias* value estimated by the gamma before the nesting effect is smaller than that of the uniform prior, and the estimation result is more stable. This shows that as the number of treatment groups decreases, the estimation results of the treatment effect and nesting effect of the gamma prior are more stable and better than those of the uniform prior.

In terms of the RMSE, the RMSE value of the treatment effect estimated by the gamma prior is similar to the uniform prior in different numbers of treatment groups. In addition, as the number of treatment groups decreases, the estimation accuracy of the nesting effect by the uniform prior has been significantly reduced, and the estimation accuracy of the gamma prior for the nesting effect has been stable at a relatively high level. Moreover, the RMSE value estimated by the gamma prior for the nesting effect is generally smaller than the RMSE value under the uniform prior condition, and the estimation result is more stable. Generally, as the number of treatment groups decreases, the gamma prior setting method performs similarly to the uniform prior in the estimation accuracy of the treatment effects and outperforms the uniform prior in the estimation accuracy of the nesting effects.

As the number of treatment groups increases, the convergence rate of the uniform prior treatment effect increases, but the convergence rate of the gamma prior treatment effect changes less in different treatment groups, and the estimation result has been stable at a higher level. Among these, the convergence rate of the gamma before the treatment effect is between 97.26 and 97.54, and the convergence rate of the uniform before the treatment effect is between 96.53 and 97.29. When the number of treatment groups is 16, the convergence rate of the uniform prior treatment effect is essentially the same as the convergence rate of the gamma prior. This shows that as the number of treatment groups increases, the gamma prior setting method performs better than the uniform prior in the convergence rate of treatment effects.

With the increase in the number of treatment groups, the convergence rate of the nesting effect under the two prior conditions has a certain downward trend in the number of different treatment groups. However, the convergence rate of the nesting effect is still stable at a relatively high level in general. Among these, the convergence rate of the gamma before the nesting effect is between 97.33 and 98.30, and the convergence rate of the uniform prior to the nesting effect is between 98.53 and 99.59. The convergence rate of the nesting effect under the two prior conditions dropped by nearly one percentage point, but the convergence rate of the uniform prior nesting effect is slightly better than that of the gamma prior in the different treatment groups. This shows that as the number of treatment groups increases, the uniform prior setting method performs better than the gamma prior in the convergence rate of the nesting effect.

As the number of treatment groups increases, the coverage of treatment effects under the two prior conditions decreases slightly, but the coverage rate of the uniform prior treatment effect is slightly better than the gamma prior performance on different treatment groups. Among them, the coverage rate of the gamma prior to the treatment effect is between 92.39 and 93.74, and the coverage rate of the uniform prior to the treatment effect is between 95.68 and 98.25. When the number of treatment groups is 16, the coverage rate of the gamma prior treatment effect rebounds slightly. This shows that as the number of treatment groups increases, the uniform prior setting method performs better than the gamma prior in the coverage of treatment effects.

As the number of treatment groups increases, the coverage of nesting effects under the two prior conditions shows a downward trend. However, the magnitude of the decrease in the gamma prior is more obvious than that of the uniform prior. Among them, the coverage rate of the gamma prior to the nesting effect is between 84.53 and 94.29, and the coverage rate of the uniform prior to the nesting effect is between 92.01 and 93.73. Under the conditions of many treatment groups, the coverage rate of the uniform prior nesting effect is higher than that of the gamma prior. Among them, when the number of treatment groups is 16, the coverage rate of the uniform prior nesting effect is 92.01, which is higher than the gamma prior of 84.53. However, under the condition that the number of treatment groups is small, the coverage rate of the gamma prior nesting effect is slightly higher than that of the uniform prior. Among them, when the number of treatment groups is 8, the coverage rate of the gamma prior nesting effect is 94.29, which is higher than the uniform prior of 93.73. This shows that under the conditions of a small number of treatment groups, the gamma prior setting method performs better than the uniform prior in the convergence rate of the nesting effect; under the conditions of many treatment groups, the gamma prior setting method used in the convergence rate of the nesting effect is not as good as the uniform prior.

With the increase in the number of treatment groups, the estimation accuracy of the bias value of the treatment effects of the two prior setting methods has been significantly improved. Moreover, the bias value of the treatment effect estimated by the gamma prior is higher than the uniform prior in different treatment groups, and the estimation result is more stable. Among them, the bias value estimated by the gamma prior for the treatment effect is between −106 and −31, and the bias value estimated by the uniform prior for the treatment effect is between −178 and −40. This shows that as the number of treatment groups increases, wasthe gamma prior setting method performs better than the uniform prior in the estimation accuracy of treatment effects.

With the increase in the number of treatment groups, the estimation accuracy of the nesting effect by the uniform prior is significantly improved, and the estimation accuracy of the nesting effect by the gamma prior is slightly decreased. However, the *bias* value estimated by the gamma before the nesting effect was smaller than that uniform prior to different treatment groups, and the estimation result was more stable. Among them, the *bias* value estimated by the gamma prior for the nesting effect is between −172 and −107, and the *bias* value estimated by the uniform prior for the nesting effect is between 120 and 1007. The *bias* value of the uniform prior estimation of the nesting effect is significantly reduced when the number of treatment groups is large, indicating that the number of treatment groups has a greater influence on the *bias* value of the uniform prior estimation of the nesting effect. Generally, as the number of treatment groups increases, the gamma prior setting method performs better than the uniform prior in the estimation accuracy of the nesting effect.

With the increase in the number of treatment groups, the estimation accuracy of the treatment effects of the two prior setting methods has been significantly improved. Moreover, the RMSE value of the treatment effect estimated by the gamma prior is similar to the uniform prior in the number of different treatment groups. Among them, the RMSE value estimated by the gamma prior to the treatment effect is between 66 and 95, and the RMSE value estimated by the uniform prior to the treatment effect is between 65 and 94. This shows that as the number of treatment groups increases, the gamma prior setting method performs similarly to the uniform prior in the estimation accuracy of treatment effects.

With the increase in the number of treatment groups, the estimation accuracy of the nesting effect by the uniform prior has been significantly improved, and the estimation accuracy of the nesting effect by the gamma prior has been stable at a relatively high level. Moreover, the RMSE value estimated by the gamma before the nesting effect is smaller than the uniform prior in different treatment groups, and the estimation result is more stable. Among them, the RMSE value estimated by the gamma prior for the nesting effect is between 30 and 31, and the RMSE value estimated by the uniform prior for the nesting effect is between 37 and 107. The RMSE value of the nesting effect estimated by the uniform prior is significantly reduced when the number of treatment groups is large, indicating that the number of treatment groups has a greater influence on the RMSE value of the nesting effect estimated by the uniform prior. Generally, as the number of treatment groups increases, the gamma prior setting method performs better than the uniform prior in the estimation accuracy of the nesting effect.

### 3.3. The Estimation Accuracy Results of Treatment Effects and Nesting Effects for the SSIND under Different Number of Subjects in Each Group

**Figure 2:**
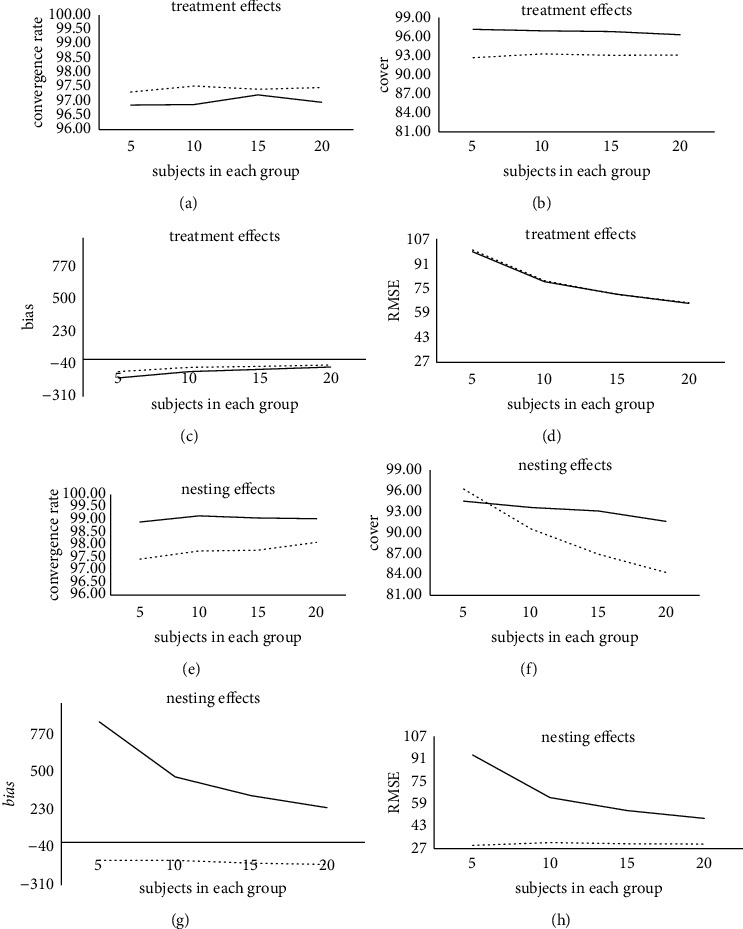
Comparison of estimation results of treatment and nesting effects for the SSIND under different number of subjects in each group. Note: the dotted line represents the estimation result of the gamma prior, and the solid line represents the estimation result of the uniform prior.

According to Tables 1–3, under the condition of a different numbers of subjects in each group, different prior setting methods have different performances on the estimation of the treatment effect and nesting effect. The result is shown in Figure 2.

As the number of subjects in each group decreases, in terms of convergence rate, the convergence rate of the gamma prior treatment effect has been stable at a relatively high level and is higher than that of the uniform prior. However, the convergence rate of the uniform prior nesting effect is higher than that of the gamma prior, and it is generally more stable. In terms of coverage, the coverage of treatment effects and nesting effects under the two prior conditions is slightly improved, but the coverage of uniform prior treatment effects and nesting effects under different treatment groups is higher and more stable overall than that of gamma priors.

In terms of *bias*, the estimation accuracy of the treatment effect of the two prior setting methods is stable at a relatively high level. However, the *bias* value when the treatment effect is estimated by the gamma prior is generally higher than the *bias* value under the uniform prior condition, and the estimation result is more stable. In addition, the estimation accuracy of the nesting effect by the uniform prior is significantly reduced, the *bias* value of the nesting effect estimation by the gamma prior is smaller than that of the uniform prior, and the estimation result is more stable. This shows that as the number of subjects in each group decreases, the estimation results of the treatment effect and nesting effect of the gamma prior are more stable and better than those of the uniform prior.

In terms of the RMSE, the RMSE value of the treatment effect estimated by the gamma prior is similar to the uniform prior in different numbers of treatment groups. In addition, as the number of subjects in each group decreases, the estimation accuracy of the nesting effect by the uniform prior was significantly reduced, and the estimation accuracy of the gamma prior for the nesting effect has been stable at a relatively high level. Moreover, the RMSE value estimated by the gamma prior for the nesting effect is generally smaller than the RMSE value under the uniform prior condition, and the estimation result is more stable. Generally, as the number of subjects in each group decreases, the gamma prior setting method performs similarly to the uniform prior in the estimation accuracy of the treatment effects and outperforms the uniform prior in the estimation accuracy of the nesting effects.

As the number of subjects in each group increases, the convergence rate of the treatment effect under the two prior conditions is slightly improved, but the convergence rate of the gamma prior treatment effect is generally higher than that of the uniform prior. Among these, the convergence rate of the gamma before the treatment effect is between 97.31 and 97.52, and the convergence rate of the uniform before the treatment effect is between 96.86 and 97.21. When the number of subjects in each group gradually increased to 15, the convergence rate of the uniform prior treatment effect steadily increased. When the number of subjects in each group increased to 20, the convergence rate of the uniform prior treatment effect suddenly decreased slightly. This shows that as the number of subjects in each group increases, the gamma prior setting method performs better than the uniform prior in the convergence rate of treatment effects.

As the number of subjects in each group increases, the convergence rate of the nesting effect under the two prior conditions is slightly improved in the number of different treatment groups, but the convergence rate of the uniform prior nesting effect is generally higher than that of the gamma prior. Among these, the convergence rate of the gamma before the nesting effect is between 97.43 and 98.10; the convergence rate of the uniform prior to the nesting effect is between 98.91 and 99.16. When the number of subjects in each group gradually increased to 10, the convergence rate of the uniform prior nesting effect reached the maximum of 99.16 and then decreased. This shows that as the number of subjects in each group increases, the uniform prior setting method performs better than the gamma prior in the convergence rate of the nesting effect.

With an increase in the number of subjects in each group, the coverage rate of the treatment effect of gamma prior increased slightly, and the coverage rate of the treatment effect under uniform prior conditions has been stable at a high level, and the upper level is higher than that of the gamma prior in different subjects. Among them, the coverage rate of the treatment effect of the gamma prior is between 92.69 and 93.29; the coverage rate of the uniform before the treatment effect is between 96.31 and 97.17. This shows that as the number of subjects in each group increases, the uniform prior setting method performs better than the gamma prior in the coverage of treatment effects.

With an increase in the number of subjects in each group, the coverage of nesting effects under the two previous conditions decreased slightly. However, the magnitude of the decrease in the gamma prior is more obvious than that of the uniform prior. Among them, the coverage rate of the gamma prior to the nesting effect is between 84.28 and 96.27, and the coverage rate of the uniform prior to the nesting effect is between 91.63 and 94.53. Under the condition that the number of subjects in each group is large, the coverage rate of the uniform prior nesting effect is higher than that of the Gamma prior. Among them, when the number of subjects in each group is 20, the coverage rate of the uniform prior nesting effect is 91.63, which is higher than the gamma prior of 84.28. However, under the condition that the number of subjects in each group is small, the coverage rate of the gamma prior nesting effect is higher than that of the uniform prior. Among them, when the number of subjects in each group is 5, the coverage rate of the gamma prior to the nesting effect is 96.27, which is higher than the uniform prior of 94.53. This shows that under the condition that the number of subjects in each group is small, the gamma prior setting method performs better than the uniform prior in the coverage of nesting effects; under the condition that the number of subjects in each group is large, the gamma prior is not as good as the uniform prior in the coverage of nesting effects.

With the increase in the number of subjects in each group, the estimation accuracy of the bias value of the treatment effect of the two prior setting methods has been significantly improved. Moreover, the bias value of the treatment effect estimated by the gamma prior is smaller than that of the uniform prior in different treatment groups, and the estimation result is more stable. Among them, the bias value estimated by the gamma prior for the treatment effect is between −102 and −47, and the bias value estimated by the uniform prior for the treatment effect is between −154 and −64. This shows that as the number of subjects in each group increases, the gamma prior setting method performs better than the uniform prior in the estimation accuracy of treatment effects.

With the increase in the number of subjects in each group, the estimation accuracy of the nesting effect by the uniform prior is significantly improved, and the estimation accuracy of the nesting effect by the gamma prior is slightly decreased. However, the bias value estimated by the gamma before the nesting effect is smaller than that of the uniform prior in different treatment groups, and the estimation result is more stable. Among them, the bias value estimated by the gamma prior for the nesting effect is between −160 and −130, and the bias value estimated by the uniform prior for the nesting effect is between 252 and 874. The bias value of the uniform prior estimation of the nesting effect effect decreases significantly when the number of subjects in each group is large, indicating that the number of subjects in each group had a greater influence on the bias value of the uniform prior estimation of the nesting effect. Generally, as the number of subjects in each group increases, the gamma prior setting method performs better than the uniform prior in the estimation accuracy of the nesting effect.

With the increase in the number of subjects in each group, the estimation accuracy of the RMSE value of the treatment effect of the two prior setting methods has been significantly improved. Moreover, the RMSE value of the treatment effect estimated by the gamma prior is similar to that of the uniform prior in the number of different treatment groups. Among them, the RMSE value of the treatment effect estimated by the gamma prior is between 66 and 100, and the RMSE value estimated by the uniform prior for the treatment effect is between 65 and 99. Under the condition that the number of subjects in each group is 20, the two prior setting methods have the smallest RMSE value for the treatment effect estimation. This shows that as the number of subjects in each group increases, the gamma prior setting method performs similarly to the uniform prior in the estimation accuracy of treatment effects.

With the increase in the number of subjects in each group, the estimation accuracy of the RMSE value of the nesting effect by the uniform prior has been significantly improved, and the estimation accuracy of the nesting effect by the gamma prior has been stable at a high level. Moreover, the RMSE value estimated by the gamma before the nesting effect is smaller than that of the uniform prior in different treatment groups, and the estimation result is more stable. Among them, the RMSE value estimated by the gamma prior for the nesting effect is stable at around 30, and the RMSE value estimated by the uniform prior for the nesting effect is between 49 and 94. The RMSE value of the nesting effect estimated by the uniform prior is significantly reduced when the number of subjects in each group is large, indicating that the number of subjects in each group has a greater influence on the RMSE value of the nesting effect estimated by the uniform prior. Generally, as the number of subjects in each group increases, the gamma prior setting method performs better than the uniform prior in the estimation accuracy of the nesting effect.

### 3.4. The Estimation Accuracy Results of Treatment Effects and Nesting Effects for the SSIND under Different ICCs

**Figure 3:**
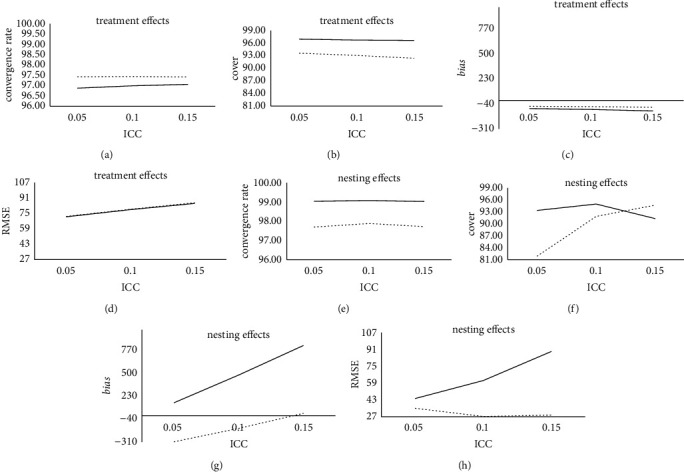
Comparison of estimation results of treatment and nesting effects for the SSIND under different treatment conditions ICC. Note: the dotted line represents the estimation result of the gamma prior, and the solid line represents the estimation result of the uniform prior.

According to Tables 1–3, under the conditions of different treatment conditions ICC, different prior setting methods have different performances on the estimation of the treatment effect and nesting effect. The result is shown in Figure 3.

The ICC under treatment conditions is also an important factor affecting the estimation accuracy of treatment effects and nesting effects parameters. With the increase of ICC under treatment conditions, in terms of convergence rate, the convergence rate of the treatment effect and nesting effect under the two prior conditions does not change significantly. However, the convergence rate of the gamma prior treatment effect is generally higher than that of the uniform prior, and the convergence rate of the gamma prior nesting effect is generally lower than that of the uniform prior. In terms of coverage, compared with the gamma prior, the coverage of treatment effects and nesting effects under uniform prior conditions does not change significantly and is stable at a relatively high level.

In terms of *bias* and RMSE, the *bias* value, and RMSE values of the treatment effect under the two prior conditions have a slight increasing trend, but in general, the *bias* value and RMSE values of the treatment effect under the gamma prior are smaller, and the performance is slightly better than the uniform prior. In addition, the *bias* value and RMSE value of the nesting effect under the uniform prior have a significant increasing trend, while the gamma prior estimating the *bias* value and the RMSE value of the nesting effect have a decreasing trend, and they are generally smaller and more stable. This shows that the carefully selected gamma prior performs better than the uniform prior.

As the ICC increases, the convergence rate of the uniform prior treatment effect increases slightly under different ICC conditions, and the convergence rate of the gamma prior treatment effect has been stable at a relatively high level. Among these, the convergence rate of the gamma before the treatment effect is between 97.42 and 97.44, and the convergence rate of the uniform prior to the treatment effect is between 96.88 and 97.06, and the overall performance is not as good as the gamma prior. This shows that with the increase of ICC, the gamma prior setting method performs better than the uniform prior in the convergence rate of treatment effects.

With the increase of ICC, the convergence rate of the nesting effect under the two prior conditions is slightly improved on different ICCs, but the convergence rate of the uniform prior nesting effect is generally higher than that of the gamma prior. Among these, the convergence rate of the gamma before the nesting effect is between 97.70 and 97.88; the convergence rate of the uniform prior to the nesting effect is between 99.03 and 99.08. When the ICC is 0.1, the two prior setting methods both perform their best, and then slightly decrease. This shows that with the increase of ICC, the uniform prior setting method performs better than the gamma prior in the convergence rate of the nesting effect.

With the increase of ICC, the coverage rate of treatment effects under the gamma prior condition decreases slightly, while the coverage rate of uniform prior treatment effects is at a higher level in different ICC and is higher than that of the gamma prior condition. Among them, the coverage rate of the gamma prior treatment effect is between 92.41 and 93.62; the coverage rate of the uniform prior treatment effect is between 96.67 and 96.98. This shows that with the increase of ICC, the gamma prior setting method is not as good as the uniform prior in the coverage of treatment effects.

With the increase of ICC, the coverage rate of the uniform prior nesting effect decreases, and the coverage rate of the gamma prior nesting effect increases significantly. Among these, the coverage rate of the gamma prior to the nesting effect is between 81.94 and 94.68, and the coverage rate of the uniform prior to the nesting effect is between 91.33 and 94.97. Under the condition of a small ICC, the coverage rate of the uniform prior nesting effect is higher than that of the gamma prior. Among them, when the ICC is 0.05, the coverage rate of the uniform prior nesting effect is 93.37, which is higher than the 81.94 of the gamma prior. However, under the condition of a large ICC, the coverage rate of the gamma prior nesting effect is higher than that of the uniform prior. Among them, when the ICC is 0.15, the coverage rate of the gamma prior nesting effect is 94.68, which is higher than 91.33 for the uniform prior. This shows that under the condition of a small ICC, the gamma prior setting method is not as good as the uniform prior in the coverage of the nesting effect; under the condition of a large ICC, the gamma prior setting method is more effective in the nesting effect.

With the increase of ICC, the estimation accuracy of the bias value of the treatment effects of the two prior setting methods has been significantly improved. Moreover, the bias value of the treatment effect estimated by the gamma prior is higher than the uniform prior in different ICC, and the estimation result is more stable. Among them, the bias value estimated by the gamma prior for the treatment effect is between −75 and −64, and the bias value estimated by the uniform prior for the treatment effect is between −115 and −89. This shows that with the increase of ICC, the gamma prior setting method performs better than the uniform prior in the estimation accuracy of treatment effects.

As ICC increases, the estimation accuracy of the bias value of the nesting effect by the uniform prior is significantly reduced, and the estimation accuracy of the nesting effect by the gamma prior is significantly improved. In addition, under the condition that the ICC is small, the uniform prior has a better estimation accuracy for the nesting effect than the gamma prior. However, under the condition of a large ICC, the estimation accuracy of the gamma prior on the nesting effect is better than that of the uniform prior, which shows that under the conditions of a large ICC, the performance of the gamma prior is better than that of the uniform prior. Among them, when ICC is 0.1 and 0.15, the bias values of the gamma prior for nesting effect estimation are −148 and 27, which are smaller than 478 and 824 of the uniform prior. Generally, with the increase of ICC, the gamma prior setting method performs better than the uniform prior under the estimation accuracy of the nesting effect.

With the increase of ICC, the estimation accuracy of the RMSE of the treatment effect of the two prior setting methods is slightly reduced. Moreover, the RMSE value of the treatment effect estimated by the gamma prior is similar to the uniform prior under different ICC conditions. Among them, the RMSE value of the treatment effect estimated by the gamma prior is between 72 and 86, and the RMSE value estimated by the uniform prior for the treatment effect is between 72 and 85. This shows that with the increase of ICC, the gamma prior setting method is similar to the uniform prior in the estimation accuracy of treatment effects.

With the increase of ICC, the estimation accuracy of the RMSE of the nesting effect by the uniform prior was significantly reduced, and the estimation accuracy of the gamma prior to the nesting effect was stable at a relatively high level. Moreover, the RMSE value estimated by the gamma before the nesting effect is smaller than the uniform prior in different ICC, and the estimation result is more stable. Among them, the RMSE value estimated by the gamma prior for the nesting effect is between 27 and 35, and the RMSE value estimated by the uniform prior for the nesting effect is between 44 and 89. The RMSE value of the nesting effect estimated by the uniform prior has a significant increase when the ICC is large, indicating that the ICC has a greater impact on the RMSE value of the nesting effect estimated by the uniform prior. Generally, with the increase of ICC, the gamma prior setting method performs better than the uniform prior in the estimation accuracy of the nesting effect.

### 3.5. Suggestions for the Use of Prior Setting for the SSIND in a Multilevel Model

This paper uses simulation research to explore the performance of gamma priors and uniform priors in estimating treatment effects and nesting effects in a multilevel model and comprehensively compares the performance of these two methods in the model on the four evaluation indicators of the convergence rate, coverage rate, *bias*, and root-mean-square error. The performance and the detailed recommendations for the use of a prior setting are shown in Table 5.

As shown in Table 5, this article recommends using the gamma prior when the number of treatment groups is small (8), and the uniform prior can be used when the number of treatment groups is large (16). When the treatment condition ICC is large (0.15), the gamma prior is used, and when the treatment condition ICC is small (0.05), the uniform prior can be used.

## 4. Conclusions and Prospects

### 4.1. Conclusion

This study adopts the Bayesian method that performs well under small sample conditions, compares the pros and cons of setting different prior distributions for the second-level nesting effect, explores the three factors that have been paid more attention to in previous studies in detail: the number of the treatment group, the number of subjects in each group, and the ICC under the treatment conditions, and explores the influence of the above factors on different parameter estimation methods, treatment effects, and nesting effect for the SSIND. The results show that for different prior setting methods, different operating conditions have a great impact; the specific conclusions are as follows.

First, when the treatment condition ICC is small (0.05), the bias and RMSE values of the parameter estimation by the gamma prior setting method are larger and the performance is unstable, while the bias and RMSE values of the parameter estimation by the uniform prior setting method are smaller and the performance is relatively stable, so the uniform prior setting method is recommended; when the treatment condition ICC is large (0.15), the bias and RMSE values of the parameter estimation by the uniform prior setting method are larger and the performance is unstable, while the bias and RMSE values of the parameter estimation by the gamma prior setting method are smaller and the performance is relatively stable, so the gamma prior setting method is recommended; when the treatment condition ICC is between 0.05 and 0.15, both prior setting methods have similar effects.

Second, when the number of treatment groups is small (8), the gamma prior setting method is recommended; when the number of treatment groups is large (16), the uniform prior setting method is recommended; when the number of treatment groups is between 8 and 16, both prior setting methods have similar effects.

Third, when we choose which prior setting method to use for the SSIND, we must consider the interaction between the ICC and the number of treatment groups.

### 4.2. Shortcomings and Prospects

Aiming at the SSIND, this study initially explored the accuracy of parameter estimation under different conditions when using a multilevel model consistent with its data structure to analyze it. The conclusions drawn verify the influence of predecessors on the number of treatment groups, the number of subjects in each group, and the treatment conditions of the ICC on the prior setting method, but there are still some shortcomings in this study, which need to be improved.

First, the SSIND discussed in this study is two-level, but in fact, there are still similar three-level data in the field of educational psychology. Future research can further explore the multilevel model to analyze the three-level small sample imbalanced nested data and explore the influence of the manipulated variables in this study on the estimation parameter accuracy of treatment effects and nesting effects.

Secondly, in this study, the number of subjects in each group under the treatment conditions is set to be equal, but in real life, there is a possibility that the number of subjects in each group will be unequal. Future research should consider setting the number of subjects in each group to be unequal and exploring the impact of different prior setting methods on the accuracy of parameter estimation results.

Finally, in view of the limited energy, other prior setting methods, such as inverse gamma priors, have not been compared in this study. I hope that researchers will conduct in-depth research on them in the multilevel model to enrich the prior setting methods of the multilevel model.

## 5. Research Significance and Innovation

### 5.1. Research Significance

From the perspective of theoretical research, this article is based on the Bayesian method that performs better under small sample conditions and solves the common maximum likelihood methods that are prone to model non-convergence when analyzing multilevel models with small-sample imbalances. This article solves the problem of estimating the parameters of the variance components outside the parameter space, and compares and analyzes the performance of setting different prior distributions for the second level of nesting effects. This article enriches the estimation methods of treatment effects and nesting effects in the multilevel model under the theoretical framework.

From the perspective of practical application, in randomized controlled experiments in clinical psychology, the SSIND is very common. For example, some alcoholic subjects were randomly assigned to the treatment group or the control group. After that, the subjects who were assigned to the treatment group were randomly assigned to several therapists to form several groups, and the participants in each group would interact with each other. In dependence, the subjects in the control group are independent of each other to explore the effect of the therapist in the process of alcohol withdrawal, that is, the treatment effect. Therefore, the research on the treatment effect estimation method in the multilevel model with a small sample imbalance has certain practical application significance. This study creates different situational conditions through simulation experiments, based on the Bayesian method that performs better under small sample conditions, and compares the performance of setting different prior distributions for the second-level nesting effect, which is proposed as a parameter prior. The setting provides reference suggestions to provide a better application plan for most researchers.

### 5.2. Research and Innovation

The innovation of this article is as follows:

First, for the SSIND, this study uses a multilevel model consistent with its data structure to analyze. At present, domestic and foreign scholars mostly ignore the imbalanced structure in the research on the small sample of imbalanced nested data and directly use the linear regression model or the traditional multilevel model to analyze. However, the use of a multilevel model consistent with this data type is critical to the accuracy of the parameter estimation results.

Second, in the selection of parameter estimation methods, this study introduces the Bayesian method into the study of multilevel models, which enriches the application scenarios of this method. In previous studies, when analyzing multilevel models with small sample imbalances, most of them used maximum likelihood or restrictive maximum likelihood estimation methods, but these methods did not perform well in the case of small samples. This study systematically evaluated the performance of different prior setting methods under the Bayesian framework in estimating the effects of a small sample imbalanced multilevel model to enrich the application of this method.

Third, when comparing the pros and cons of different prior setting methods, this research conducted analysis and comparison with more conditions. Previous studies mostly focused on analysis under a small number of conditions, but this study more systematically explored the performance of several different prior distribution methods in 36 condition combinations composed of the number of treatment groups, the number of subjects in each group, and the treatment conditions.

Fourth, when measuring the performance of different prior setting methods, this study broadens the selection range of evaluation indicators. Most previous studies only considered convergence rate, deviation, or root-mean-square error as evaluation indicators, but this study uses four indicators of convergence rate, confidence interval coverage, treatment effects/nesting effects estimation deviation, and root-mean-square error. In this aspect, the performance of the gamma prior and the uniform prior distribution methods is comprehensively compared, and the applicability and effectiveness of the two prior distribution methods in the multilevel model with small sample imbalance are systematically evaluated.

## Figures and Tables

**Table 1:** Estimated accuracy results of treatment effects and nesting effects for the SSIND under all operating conditions (*c* = 8).

The number of subjects in each group	ICC	Prior setting	Convergence rate		Cover		Bias		RMSE	
			Treatment	Nesting	Treatment	Nesting	Treatment	Nesting	Treatment	Nesting
			Effect	Effect	Effect	Effect	Effect	Effect	Effect	Effect
5	0.05	U	96.1	98.8	98.02	94.94	−245	1338	113	134
		G	97.6	97.9	93.75	97.55	−171	−186	114	27
	0.10	U	97	99.6	98.56	94.38	−272	1656	118	166
		G	97.8	98.4	93.76	97.76	−156	−96	121	27
	0.15	U	96.7	99.4	98.24	91.65	−301	1979	123	198
		G	97.1	97.6	92.07	96.72	−169	−3	125	29

10	0.05	U	96	99.6	98.44	95.78	−177	582	88	65
		G	97.3	98.7	95.17	95.85	−114	−114	88	32
	0.10	U	96.9	99.5	98.14	95.18	−193	982	97	102
		G	97.8	98.3	93.76	96.64	−103	−120	98	29
	0.15	U	96.7	99.5	98.35	89.15	−194	1433	104	146
		G	98	98.1	92.86	96.94	−105	33	106	32

15	0.05	U	96.3	99.9	99.17	96.4	−121	309	73	45
		G	97.7	97.7	94.68	90.79	−86	−303	73	33
	0.10	U	97.2	100	98.87	96.2	−145	767	84	84
		G	97	98.4	94.43	94.11	−108	−119	84	27
	0.15	U	96.3	99.8	98.44	89.18	−190	1242	93	128
		G	96.3	98.8	94.08	94.43	−100	59	93	30

20	0.05	U	96.9	99.6	97.94	96.89	−84	138	68	37
		G	98	98.2	94.18	84.52	−40	−334	68	36
	0.10	U	96.6	99.8	97.52	95.29	−88	597	79	70
		G	98.1	99.1	94.09	92.03	−55	−142	79	28
	0.15	U	96.5	99.6	97.31	89.76	−120	1061	88	112
		G	97.8	98.4	92.02	94.11	−68	44	88	30

**Table 2:** Estimated accuracy results of treatment effects and nesting effects for the SSIND under all operating conditions (*c* = 12).

The number of subjects in each group	ICC	Prior setting	Convergence rate		Cover		Bias		RMSE	
			Treatment	Nesting	Treatment	Nesting	Treatment	Nesting	Treatment	Nesting
			Effect	Effect	Effect	Effect	Effect	Effect	Effect	Effect
5	0.05	U	96.9	98.8	97.42	96.66	−148	385	90	47
		G	96.8	97.1	93.49	96.19	−132	−253	91	31
	0.10	U	97.3	99.3	97.64	95.87	−139	623	94	67
		G	98.2	97.9	93.58	97.96	−102	−137	94	28
	0.15	U	96.4	98.9	96.99	91.41	−153	899	98	93
		G	97.5	97.2	91.49	97.12	−125	−14	100	30

10	0.05	U	96.7	98.9	96.28	96.66	−72	−3	71	30
		G	98.1	97.5	92.97	84.72	−55	−326	71	36
	0.10	U	96.6	99.1	96.38	94.35	−80	315	77	46
		G	97.8	97.6	91.62	93.24	−59	−154	78	29
	0.15	U	97	99.2	95.77	91.03	−93	641	83	72
		G	97.1	98.3	92.79	94.51	−72	31	83	31

15	0.05	U	98.2	99.5	97.15	96.28	−44	−120	61	27
		G	97.7	98.2	92.73	76.48	−26	−354	61	37
	0.10	U	97.3	99	96.4	95.66	−39	204	70	38
		G	96.9	98.2	92.88	89.21	−32	−152	69	27
	0.15	U	97.3	98.9	96.61	91.61	−52	551	77	63
		G	97.6	96.7	91.39	94.31	−43	51	77	28

20	0.05	U	96.3	98.9	96.16	90.6	−44	−193	56	28
		G	97.2	97.4	92.7	69.82	−51	−375	57	38
	0.10	U	96.8	98.7	95.45	94.33	−62	145	66	34
		G	97.5	97.9	91.79	89.17	−60	−160	66	25
	0.15	U	97.6	99	95.7	92.63	−90	490	74	57
		G	97.4	98.1	91.27	93.07	−64	49	74	26

**Table 3:** Estimated accuracy results of treatment effects and nesting effects for the SSIND under all operating conditions (*c* = 16).

The number of subjects in each group	ICC	Prior setting	Convergence rate		Cover		Bias		RMSE	
			Treatment	Nesting	Treatment	Nesting	Treatment	Nesting	Treatment	Nesting
			Effect	Effect	Effect	Effect	Effect	Effect	Effect	Effect
5	0.05	U	97.7	98.5	96.21	97.46	−45	105	80	30
		G	96.5	96.8	92.33	92.67	−6	−285	80	33
	0.10	U	97	98.5	95.88	96.04	−42	314	84	44
		G	96.5	97	92.12	94.23	−31	−171	84	29
	0.15	U	96.6	98.4	95.55	92.38	−43	563	88	64
		G	97.8	97	91.62	96.19	−23	−26	89	30

10	0.05	U	96.6	99	96.79	93.03	−23	−159	59	28
		G	96.9	97.4	94.22	73.1	−25	−361	60	38
	0.10	U	97.6	98.9	96.41	95.55	−35	102	65	32
		G	97.2	96.8	93.31	87.3	−25	−178	64	28
	0.15	U	97.8	98.7	95.91	91.79	−42	387	70	50
		G	97.5	97.1	92.92	92.48	−43	16	71	27

15	0.05	U	97.3	98.1	94.66	85.52	−37	−257	54	30
		G	97.9	97.2	92.65	64	−39	−391	54	40
	0.10	U	97.3	98.1	94.45	94.7	−54	28	61	28
		G	98.1	97.2	92.05	85.49	−40	−178	61	26
	0.15	U	97.7	98.3	95.5	92.47	−67	329	67	44
		G	97.5	97.7	92.82	93.14	−57	36	67	26

20	0.05	U	97.5	98.8	95.49	80.16	−25	−291	45	32
		G	97.4	98.3	94.56	57.58	−21	−398	46	40
	0.10	U	96.3	98.4	95.53	92.07	−30	5	52	26
		G	96.4	97.8	93.88	85.07	−33	−172	53	25
	0.15	U	98.1	98.6	95.72	92.9	−32	313	59	41
		G	97.4	97.7	93.53	93.14	−28	46	60	25

**Table 4:** Summary of estimation accuracy of treatment effects and nesting effects for the SSIND under all operating conditions.

	Prior setting	Convergence rate			Cover			Bias			RMSE		
		Treatment effect	Nest effect		Treatment effect	Nest effect		Treatment effect	Nest effect		Treatment effect	Nest effect	
Mean value	U	97.0		99.0	96.81		93.22	−101	485		79	65	
	G	97.4		97.8	93.04		89.49	−69	−143		79	30	

Median value	U	96.9		99.0	96.51		94.34	−76	357		77	47	
	G	97.5		97.8	92.90		93.14	−56	−147		78	29	

Minimum value	U	96.0		98.1	94.45		80.16	−301	−291		45	26	
	G	96.3		96.7	91.27		57.58	−171	−398		46	25	

Maximum value	U	98.2		100.0	99.17		97.46	−23	1979		123	198	
	G	98.2		99.1	95.17		97.96	−6	59		125	40	

*Note.* “G” means gamma prior, and “U” means uniform prior.

**Table 5:** Summary table of applicable conditions for a prior setting for the SSIND.

Condition combination	ICC = 0.05			ICC = 0.10			ICC = 0.15		
	*c* = 8	*c* = 12	*c* = 16	*c* = 8	*c* = 12	*c* = 16	*c* = 8	*c* = 12	*c* = 16
*m* = 5	G	U	U	G	G	U	G	G	G
*m* = 10	G	U	U	G	G	U	G	G	U
*m* = 15	G	U	U	U	G	U	G	G	G
*m* = 20	U	U	U	G	U	U	G	G	G

*Note.* “G” means that gamma performs better, and “U” means that uniform performs better.

## Data Availability

The data that support the findings of this study are only available on request from the corresponding author. The data are not publicly available due to privacy or ethical restrictions.
